# Test-retest reliability of an insole plantar pressure system to assess gait along linear and curved trajectories

**DOI:** 10.1186/1743-0003-11-95

**Published:** 2014-06-05

**Authors:** Marco Godi, Anna Maria Turcato, Marco Schieppati, Antonio Nardone

**Affiliations:** 1 Posture and Movement Laboratory, Division of Physical Medicine and Rehabilitation, Scientific Institute of Veruno (NO), Fondazione Salvatore Maugeri (IRCCS), Veruno, (NO), Italy; 2 Department of Translational Medicine, University of Eastern Piedmont, Novara, Italy; 3 Centro Studi Attività Motorie (CSAM), Fondazione Salvatore Maugeri (IRCCS), Scientific Institute of Pavia, Pavia, Italy; 4 Department of Public Health, Experimental and Forensic Medicine, University of Pavia, Pavia, Italy

**Keywords:** Plantar pressure system, Reliability, Gait, Curved trajectories

## Abstract

**Background:**

Previous studies have assessed reliability of insole technology for evaluating foot pressure distribution during linear walking. Since in natural motion straight walking is intermingled with turns, we determined the test-retest reliability of insole assessment for curved as well as linear trajectories, and estimated the minimum number of steps required to obtain excellent reliability for each output variable.

**Methods:**

Sixteen young healthy participants were recruited. Each performed, two days apart, two sessions of three walking conditions: linear (LIN) and curved, clockwise (CW) and counter-clockwise (CCW). The Pedar-X system was used to collect pressure distribution. Foot print was analyzed both as a whole and as subdivided into eight regions: medial and lateral heel, medial and lateral arch, I metatarsal head, II-V metatarsal heads, hallux, lateral toes. Reliability was assessed by using intraclass correlation coefficient (ICC) for clinically relevant variables from analysis of 50 steps per trajectory: Peak Force (PF); Peak Pressure (PP); Contact Area (CA); Stance Duration (S).

**Results:**

When considering whole-foot, all variables showed an ICC >0.80, therefore highly reliable. This was true for both LIN and curved trajectories. There was no difference in ICC of the four variables between left and right foot. When collapsing foot and trajectories, S had a lower ICC than PP and CA, and PP lower than CA. Mean percent error between the values of first and second session was <5%. When separately considering the eight foot regions, ICCs of PF, PP and CA for all regions and trajectories were generally >0.90, indicating excellent reliability. In curved trajectories, S showed smaller ICCs. Since the least ICC value for S was 0.60 in LIN trajectory, we estimated that to achieve an ICC ≥0.90 more than 200 steps should be collected.

**Conclusions:**

High reliability of insole dynamic variables (PF, PP, CA) is obtained with 50 steps using the Pedar-X system. On the contrary, high reliability of temporal variable (S) requires a larger step number. The negligible differences in ICC between LIN and curved trajectory allow use of this device for gait assessment along mixed trajectories in both clinical and research setting.

## Background

Measurement of plantar pressure is commonly used in clinical evaluation of the foot function during activities such as walking or running [1,2] and has proven useful to detect foot pathologies [3-6]. Plantar pressure analysis has advanced knowledge on foot loading and foot deformity during gait in diabetic neuropathy [7]. It has also been used in spinal cord injury patients to record temporal gait parameters [8], and in stroke patients to assess the effects of specific rehabilitation exercises [9,10]. Contact area and peak force were the most responsive variables in detecting changes after rehabilitation [10,11].

Reliability of insole plantar pressure measures is paramount for an accurate result, and high test-retest reliability of insole devices during linear walking is well documented [3,12]. But natural locomotion includes, besides linear walking, also frequent turns and curved trajectories [13-15]. These involve a complex reorientation of head, trunk, pelvis and feet, and are accompanied by postural adjustments to counteract the centrifugal acceleration acting on the body, as well as asymmetric motion of the lower limb, whereby the leg inside the trajectory travels a shorter pathway than the outside leg [14,16]. Not unexpectedly, recent studies requiring subjects to travel both linear and circular trajectories have detected abnormalities in patients with neurological disorders. Both Parkinson’s disease [17] and stroke patients [18] showed more severe walking difficulties during circular than linear trajectories.

Therefore, foot action during curved walking merits investigation. Knowledge of the pattern of distribution of pressures during curved walking [19] may be useful for comparing healthy subjects with patients, for detecting changes due to central or peripheral nervous system diseases, or for estimating the evolution of the gait disorder and the potential advantage of rehabilitation. To our knowledge, no study has assessed the reliability of insole technology during non-linear walking, as a premise for dependable assessment of plantar pressure measures during gait.

Advantages of using insole technology are: it avoids the need to walk exactly over a predetermined course [20] to measure pressure, force and contact area from each single foot independently, and to acquire several steps within each walking trial rather than just 1–2 steps, as with force platforms. This information is critical for assessing gait quality. Nonetheless, insole systems have some drawbacks normally affecting linear walking that may also apply, *a fortiori*, to curved walking, e.g. sensor migration due to shear stress at the foot-shoe interface and deformation of the insoles [20,21]. These mechanical phenomena might affect accurate detection of the vertical projection of the forces on the sensors, leading to different values of the vertical force with respect to the values recorded by a force platform. Also, insoles measure the force perpendicular to each sensor in the matrix, which is not necessarily identical, even if very close to the ground reaction force during curved walking [21-23]. These considerations prompted the present investigation on the test-retest reliability of insole plantar devices in curved vs. linear walking. For minimizing variability connected with frequently varying foot orientation at stance, anticipation of curvature, and replication of several separate trails, a mere circular trajectory was chosen that allowed collection of numerous similar steps, instead of a more complex path such as a figure of 8 path [24]. In addition, we aimed to quantify the steps necessary for reaching a reliable insole assessment during gait, since this is not yet fully established. It was reported that 400 strides are required to accurately measure stride variability during treadmill locomotion [25], whereas others [26] claimed that as few as 5 to 8 strides can give a reliable measure of gait parameters during linear walking. We do not know whether such figures also apply to curved walking.

## Methods

### Subjects

Sixteen healthy participants (9 women, 7 men), aged 21–35 years (mean age 25.1 ± 4.3 SD), mean Body Mass Index 22.1 ± 2.8 SD, were recruited. No subject had history of neurological diseases. All were free from ankle or foot pathology. Exclusion criteria were any major trauma in the last 6 months or lower limb surgery at any time previously. The study was approved by the Central Ethics Committee of the Fondazione Salvatore Maugeri and all subjects gave their informed consent.

### Procedure

Each participant was tested twice, with a 2–4 day interval between sessions. In both sessions, subjects walked under three different conditions: linear walking (LIN) and curved walking: clockwise (CW) and counter-clockwise (CCW). The conditions were randomized across sessions and subjects. The circle trajectory (1.2 m radius) was designed with a continuous tape stuck on the floor. This radius length had been used before, and proven adequate for highlighting the peculiar features of curved walking [14-16]. For each session, subjects executed two 20 m length trials for each trajectory, making a total of 6 trials. Before data acquisition, each subject performed two short practice trials for each condition to familiarize with instrumentation and task. Subjects were instructed to walk looking forward, head erect, without gazing constantly to the tape but walking along it as smoothly as possible. Conditions, walking instructions and operator were the same in the two sessions: every effort was made to reduce variability (e.g. laboratory setting, time of day).

### Data collection and treatment

Walking time was monitored using photocells to assure consistent stopping of the stopwatch. For LIN trajectory, photocells were placed at the beginning and at the end of the 20 m pathway, whilst for curved trajectories photocells were placed at the beginning of the first lap and at the end of the third lap. The first and last two steps of each trial were not included in the acquisition, because changes in spatial-temporal gait variables are known to occur at initiation and termination of a walk [27]. The analysis was performed on 50 steps. In all subjects, both feet were instrumented.

**Figure 1:**
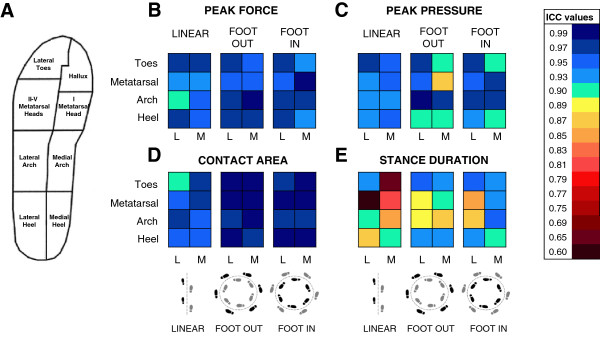
**Test-retest repeatability of each of the eight anatomical regions of the foot (A).** Intraclass correlation coefficients (ICC) obtained from 50 consecutive steps performed under each trajectory (linear; CW, clockwise; CCW, counter-clockwise) for the 4 output variables (peak force, **B**; peak pressure, **C**; contact area, **D**; stance duration, **E**). In the bottom insets, the grey foot is the right foot; this is the Foot-In for CW, and the Foot-Out for CCW. ICC values are obtained from the average of left and right foot in the case of linear trajectory, and Foot-In or Foot-Out in the case of CW and CCW. Each colour corresponds to a range of ICC value equal to ± 0.02 around the depicted mean value. L, lateral region of foot; M, medial region of foot.

We used the foot insoles Pedar-X system®, produced by Novel (Germany), which we currently use in our patients for assessing the distribution of the ground reaction forces during the stance phase of gait. These insoles are considered to accurately measure forces and exhibit low sensor to sensor random errors [28,29]. All subjects wore sneakers of the same type (Superga® 2750 model, Italy) corresponding to the individual’s size. The insoles were placed inside the shoes and connected to the Pedar box: subjects wore no socks. The insoles were calibrated using the proprietary calibration device according to the manufacturer’s manual. Data were sampled at 50 Hz. Since the trajectories could differently influence the spatial-temporal pattern of activation of different parts of the foot as well as the sensor activation, to assess repeatability across the entire foot print, the output of all 99 insole sensors was clustered into eight separate anatomical regions: medial and lateral heel, medial and lateral arch, I metatarsal head, II-V metatarsal heads, hallux, lateral toes [5] (Figure 1A). The Pedar software computes several dynamic and temporal variables. The variables chosen here were four of the most clinically significant [3]: Peak Force (N); Peak Pressure (kPa); Contact Area (cm^2^); Stance Duration (s). Peak Force and Peak Pressure were respectively the maximum force and pressure that occurred in one single sensor during the whole stance phase. Contact Area was the maximum area of active sensors during the whole stance phase.

### Estimation of ICC and Fisher’s z-scores

The average value of each of the 4 output variables for each anatomical region and foot and subject was computed from the 50 steps. This value was used for the repeatability calculation for each output variable and foot and trajectory. The reliability was estimated by means of the intraclass correlation coefficient (ICC) between 16 pairs of observations. In particular we used an ICC 3,k type (Model 3, Form k) [30] because the rater was fixed and the data used to calculate the ICC were the mean data from two walking trials performed by the 16 subjects in the two sessions.

Statistical analysis was conducted using SPSS software (Chicago, USA). We selected the option “Absolute agreement” in order to take into account the systematic error between the two sessions [31]. SPSS calculates the ICC from the two-way ANOVA result [32], with the following formula:

(1) $$\mathrm{ICC}\;\text{agreement}=\frac{\left(\mathrm{BMS}-\mathrm{EMS}\right)}{\mathrm{BMS}+\frac{\left(\mathrm{OMS}-\mathrm{EMS}\right)}{n\;\text{patients}}}$$

where BMS is the Mean Square between subjects, OMS is the Mean Square between observers and EMS is the Mean Square error.

For each output variable and trajectory, we calculated the magnitude of error that one can expect with the insole system. From the absolute mean error (the difference between the values of the first and the second day of acquisition for each variable), we computed the percent mean error (% Err) by normalizing each absolute mean error to the relevant values measured in the first day of acquisition. We also calculated the minimum detectable change (MDC) which represents the smallest change in the variable that likely reflects true change rather than measurement error alone. The calculation is the result of the multiplication of the standard error of measurement (SEM) × z value × √2. The 95% confidence level (MDC_95_) was then established.

Comparison of the output variables of each subject between the two sessions gave a total of 192 ICCs (4 output variables × 8 foot regions × 2 feet × 3 walking conditions). The repeatability was assessed, for each variable, by contrasting the two sessions of 16 subjects. The ICC values were Fisher’s z-transformed, in order to statistically assess whether the ICCs were different across variables or walking conditions. The Fisher’s z-values were then back-transformed to ICC values [33] for illustration purposes.

In addition, for each output variable we averaged the z-transform values of the 8 foot regions (this value was named ‘whole-foot’ z-score) separately for LIN, CW and CCW conditions. This was done to test, by means of parametric statistical analysis, the hypothesis that no difference in the repeatability was present between left and right ‘whole-foot’ for each variable under the three walking conditions. This calculation led to 24 ‘whole-foot’ z-scores (4 output variables × 2 feet × 3 trajectories).

### Comparison of z-scores and ICCs

We performed an analysis of variance (ANOVA) to assess differences in ‘whole foot’ z-score between the variables (Peak Force, Peak Pressure, Contact Area, Stance Duration), between feet, and between walking conditions (LIN, CW, CCW). Therefore, the 3-way ANOVA considered 4 output variables, 2 feet, 3 walking conditions as independent measures and z-score values as dependent variables. During LIN, the ANOVA on each ‘whole-foot’ z-score was run between right and left foot. During curved trajectory, meaningful functional comparisons between feet were made taking into account the position of the foot with respect to the trajectory, i.e. inside (Foot-In) or outside (Foot-Out). Therefore, we compared the ‘whole-foot’ z-score of Foot-In during CW, corresponding to the right foot, with the Foot-In during CCW, corresponding to the left foot. ‘Whole-foot’ z-score comparison for Foot-Out was done analogously.

To assess differences in gait speed between the two sessions and under the three conditions, we performed a 2-way ANOVA (2 sessions and 3 trajectories as independent measures and gait speed as dependent variable). For all ANOVAs, the Tukey’s post-hoc test was used for relevant comparisons between variables.

### Estimation of the effect of the number of steps on the ICC analysis

Since the ICC value depends, among other things, on the number of steps entering the analysis, and since not all output variables may have a very high ICC value, we separately estimated the ICC values for a variable number of steps. To this end, we estimated the minimum number of steps required to obtain a reliability of at least 0.9 of each output variable by means of the Spearman-Brown prediction formula, in which the ICC obtained in the 50-step trials was entered. For the sake of simplicity, we restricted the analysis to those foot regions displaying the lowest ICC for each variable, regardless of the trajectory.

## Results

Table 1 shows the values of gait speed for all subjects and trajectories in the two sessions used for the reliability calculation. As already shown [17,18], gait speed was slower for curved than LIN trajectory (2-way ANOVA, F(2,90) = 44.78, p = 0.0001). The post-hoc test showed no difference in gait speed either between CCW and CW or between the two sessions (F(2,90) = 0.01, p = 0.23). For all subjects, velocities did not vary more than 12% between the two sessions [34]. The coefficient of variation was small, and exhibited minor changes between trajectories and sessions, ranging from 0.06 during LIN trajectory (2^nd^ session) to 0.15 during CCW (1^st^ session).

**Table 1:** Gait speed (m/s) of single subjects during the two sessions on the three different trajectories

**Subjects**	**1st session**			**2nd session**		
	**LIN**	**CCW**	**CW**	**LIN**	**CCW**	**CW**
**1**	1.54	1.33	1.19	1.44	1.20	1.18
**2**	1.46	1.38	1.40	1.59	1.42	1.44
**3**	1.62	0.95	1.01	1.64	1.00	1.03
**4**	1.80	1.57	1.59	1.67	1.27	1.29
**5**	1.30	1.36	1.20	1.51	1.45	1.30
**6**	1.44	1.16	1.19	1.54	1.29	1.27
**7**	1.45	1.29	1.39	1.47	1.42	1.47
**8**	1.62	1.03	1.22	1.57	1.16	1.30
**9**	1.53	1.24	1.23	1.57	1.29	1.28
**10**	1.52	1.20	1.16	1.56	1.23	1.18
**11**	1.70	1.56	1.40	1.74	1.58	1.44
**12**	1.58	1.00	1.01	1.62	1.23	1.22
**13**	1.32	1.01	0.99	1.36	1.04	1.02
**14**	1.46	1.15	1.14	1.50	1.19	1.18
**15**	1.50	1.23	1.21	1.54	1.27	1.25
**16**	1.52	1.25	1.23	1.55	1.30	1.25
**Mean**	1.52	1.23	1.22	1.55	1.27	1.26
**SD**	0.13	0.18	0.16	0.09	0.15	0.13
**CV%**	0.08	0.15	0.13	0.06	0.12	0.10

LIN, linear trajectory; CCW, counterclockwise trajectory; CW, clockwise trajectory; SD, standard deviation; CV, coefficient of variation.

### Reliability of the insole output variables between left and right foot for the three trajectories

Table 2 shows the ICC values of each ‘whole-foot’ (and the 95% Confidence Interval) for the three trajectories and for the four output variables as well as the respective percent mean error (% Err) between the values of the first and second day of acquisition. Percent Err was only 4.8% (average from all output variables of left and right foot during the three trajectories). Further, there were no differences in the ICC of the four insole output variables (Peak Pressure, Peak Force, Contact Area and Stance Duration) between left and right foot, either during LIN or curved trajectory (z-score, 3-way ANOVA, F(6,168) = 0.32, p = 0.93) (Table 3). In spite of overall high z-score values, there were small but significant differences in ICC between trajectories. ‘Whole-foot’ z-score was 1.76 (ICC = 0.94), 2.05 (ICC = 0.96), and 2.1 (ICC = 0.97) for LIN, Foot-In, Foot-Out (3-way ANOVA, F(2,168) = 15.36, p < 0.0001).

**Table 2:** Summary of reliability results of each ‘whole-foot’ for the three walking trajectories and for the four output variables

	**Left**				**Right**			
	**ICC**	**CI 95%**	**% Err**	**MDC**_**95**_	**ICC**	**CI 95%**	**% Err**	**MDC**_**95**_
**Linear trajectory**								
Peak Force	0.96	(0.94-0.97)	4.16	82.71 (N)	0.96	(0.93-0.97)	4.28	85.39 (N)
Peak Pressure	0.95	(0.93-0.97)	6.73	4.23 (kPa)	0.95	(0.93-0.97)	3.81	3.11 (kPa)
Contact Area	0.96	(0.94-0.98)	4.92	9.38 (cm^2^)	0.96	(0.94-0.98)	4.64	10.38 (cm^2^)
Stance	0.81	(0.62-0.89)	4.62	0.05 (s)	0.87	(0.80-0.92)	3.41	0.04 (s)
**Clockwise trajectory**								
Peak Force	0.97	(0.95-0.99)	4.47	67.84 (N)	0.97	(0.95-0.98)	3.60	68.87 (N)
Peak Pressure	0.97	(0.93-0.98)	8.75	3.00 (kPa)	0.95	(0.91-0.97)	6.78	4.3 (kPa)
Contact Area	0.99	(0.98-1.00)	3.76	5.07 (cm^2^)	0.99	(0.98-1.00)	2.95	5.63 (cm^2^)
Stance	0.93	(0.89-0.95)	4.43	0.05 (s)	0.92	(0.87-0.95)	3.47	0.06 (s)
**Counterclockwise trajectory**								
Peak Force	0.97	(0.95-0.99)	4.65	66.56 (N)	0.97	(0.96-0.98)	4.33	66.21 (N)
Peak Pressure	0.96	(0.93-0.98)	5.59	3.37 (kPa)	0.94	(0.87-0.97)	10.14	3.2 (kPa)
Contact Area	0.99	(0.97-0.99)	3.95	5.23 (cm^2^)	0.99	(0.97-1.00)	3.50	5.29 (cm^2^)
Stance	0.93	(0.88-0.96)	4.46	0.05 (s)	0.91	(0.85-0.94)	4.86	0.05 (s)

ICC, intraclass correlation coefficient; CI 95%, 95% Confidence Interval of ICC; % Err, percentage mean error between values of the first and the second day of acquisition; MDC_95_, minimum detectable change at the 95% confidence level.

**Table 3:** 3-way ANOVA of each ‘whole-foot’: 2 feet (left-right) × 3 trajectories × 4 output variables

	**F**	**df**	**P**
Feet	0.01	1,168	0.91
Trajectories	15.36	2,168	<0.0001
Output variables	52.85	3,168	<0.0001
Feet × trajectories	0.33	2,168	0.71
Output variables × feet	0.87	3,168	0.45
Output variables × trajectories	2.67	6,168	0.02
Output variables × feet × trajectories	0.32	6,168	0.93

There was a statistically significant difference in the z-scores of the ICC calculated for the four output variables (trajectory and foot collapsed) (F(3,168) = 52.85, p < 0.0001). There was an interaction between variables and trajectories (F(6,168) = 2.67, p < 0.02), due to the relatively lower z-score value of the variable Stance Duration during LIN than CW or CCW (post-hoc, p < 0.0005). The z-score of Foot-In and Foot-Out were not different for Stance Duration (p > 0.05), regardless of the trajectory. However, during curved trajectory, Stance Duration had a lower z-score than Peak Pressure and Contact Area (p < 0.001), and in turn Peak Pressure had a lower z-score than Contact Area (p < 0.0001). For Peak Force, the post-hoc test showed no difference in the z-scores between trajectories (p > 0.05 for all comparisons). All other interactions between output variables, feet and trajectories were not significant.

Table 2 also shows the minimum detectable change at the 95% confidence level (MDC_95_), which represents the smallest change in score that likely reflects true change rather than measurement error alone. The highest values were found for Peak Force and the lowest for Stance Duration, because the MDC values is proportional to the scale of the original variable.

### Reliability across foot anatomical regions

Figure 1 summarizes in a graphical form the ICCs of the eight foot regions and variables in the three walking conditions. For the sake of simplicity, given that no ICC difference had been previously detected between feet, the data of each anatomical region from the two feet were averaged.The ICCs of Peak Force for all foot regions and trajectories were above 0.90, indicating excellent reliability (Figure 1B). For instance, ICCs of Peak Force for LIN ranged from 0.92 (lateral arch) to 0.98 (lateral heel). For Foot-In and Foot-Out in curved trajectory, ICC ranged from 0.95 (II-V metatarsal heads) to 0.99 (medial arch), and from 0.94 (hallux) to 0.99 (I metatarsal head). The ICCs of Peak Pressure for each region and trajectory were above 0.90 except for hallux of Foot-Out (Figure 1C). ICCs of Peak Pressure for LIN ranged from 0.93 (II-V metatarsal heads) to 0.98 (toes). For Foot-In and Foot-Out in curved trajectory, the ICC of Peak Pressure ranged respectively from 0.90 (II-V metatarsal heads) to 0.98 (I metatarsal head) and from 0.88 (hallux) to 0.99 (lateral arch). The ICCs of Contact Area for each foot region and trajectory considered were above 0.90 (Figure 1D), ranging from 0.90 (toes) to 0.98 (hallux) in LIN, from 0.97 (II-V metatarsal heads) to 0.99 (lateral heel) for Foot-In and from 0.88 (medial heel) to 0.97 (lateral toes) for Foot-Out.The ICCs for Stance Duration were below 0.90 in LIN trajectory for all foot regions (range 0.60-0.87 for II-V metatarsal heads and medial heel, respectively), indicating moderate to good reliability (Figure 1E). In curved trajectory, Stance Duration showed ICCs ranging from 0.85 (II-V metatarsal heads) to 0.95 (lateral heel) for Foot-In and from 0.86 (lateral arch) to 0.96 (lateral toes) for Foot-Out.

### Number of steps required to achieve a minimum ICC value of 0.90 in all regions of the foot

**Figure 2:**
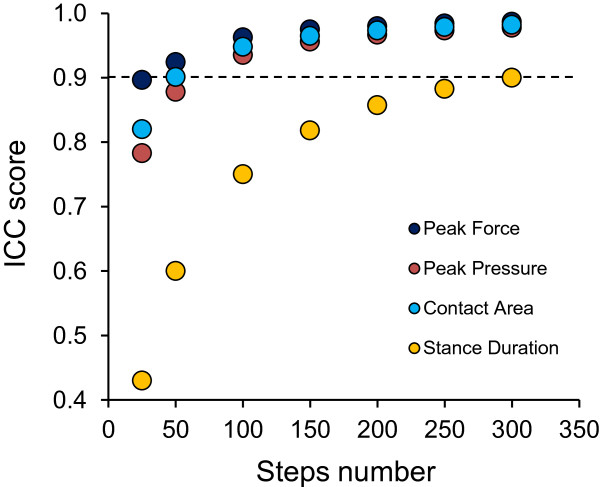
**Number of steps required to reach an ICC of 0.90 (good reliability) for the four variables considered.** In the case of Peak Force, 25 steps are sufficient. For Stance Duration, it is necessary to collect more than 200 steps.

The above reported findings were obtained by analyzing the output variables collected during 50 steps. We then estimated the number of steps needed to achieve an ICC of at least 0.90 in the foot region exhibiting the lowest ICC value in the standard trial of 50 steps. In turn, the chosen trajectory was that exhibiting the lowest ICC values. This was done for all four variables, by using the Spearman-Brown prediction formula.Figure 2 shows the results of the estimate. As an example, to achieve an ICC value for Peak Pressure higher than 0.90 in the least reliable foot region (the lowest ICC at 50 steps for Peak Pressure was found at the medial heel region in Foot-Out), at least 65 steps should be collected. More than 200 steps should instead be collected in order to achieve an ICC for Stance Duration of about 0.90. On the other hand, for Peak Force, an ICC of 0.90 could be achieved with only 25 steps.

## Discussion

This study was prompted by the need to assess the reliability of pressure insoles as a tool for evaluating gait improvement from rehabilitation. If plantar pressure analysis is to be used to test the effect of a treatment, it is necessary to consider values of test-retest reliability based on different sessions. Given that the effect of rehabilitation may be small in certain diseases, or builds up slowly over successive training sessions, a reliable measuring instrument and procedure can provide insight into whether improvement is occurring. Moreover, since gait rehabilitation must address conjointly linear and curved walking, accurate measurement of both tasks is necessary. Therefore, this study assessed for the first time the reliability of an insole plantar pressure system to evaluate gait during curved walking. We also considered the output variable Stance Duration, while most other studies have limited their analysis to Peak Force, Peak Pressure and Contact Area [5].

For clinical purposes, ICC values should exceed 0.90 to ensure reliability [30]. Previous studies have demonstrated that pressure, force and area can be reliably assessed using insole technology [3,5,35] during linear walking. Our study confirms the high test-retest reliability for the Pedar-X system analysis of foot loading and extends this notion to curved trajectories. The analysis was based on a virtual ‘whole-foot’, and extended to each of eight distinct anatomical foot regions.

In our study, Peak Force, Peak Pressure and Contact Area showed excellent reliability for a 50-step locomotor task along the three trajectories. A previous study showed that mid-foot region and plantar arch have low reliability because of the reduced pressure and force applied on those regions during walking [7]. Our data are instead in keeping with Murphy et al. [35] and Ramanathan et al. [5], who found that Contact Area and Plantar Pressure data revealed excellent reliability in straight walking also in the mid-foot region. Conversely, we found a low reliability for Stance Duration, in accordance with Putti et al. [3]. Reliability of Stance Duration was low for straight and somewhat better for curved walking. In order to achieve a high reliability for Stance Duration in all 8 foot regions and in the three walking conditions, more than 200 steps should be collected. Notably, Peak Pressure and Contact Area require less numerous steps (50 steps), and the estimated step number for Peak Force was even smaller (25 steps). In a sense, the reliability of pressure insoles output is inferior to that of other devices such as baropodometric walkways [26] specifically designed for assessment of kinematic variables such as Stance Duration. More than 200 steps may be in fact close to or over the limit of a walking test for some patients. However, plantar pressure analysis bypasses the constraints connected to other baropodometric devices like mats, requiring straight walking, or force platforms that record one foot placement at a time and prevent any reliable assessment of curved walking.

We cannot readily explain why Stance Duration ICC was generally weaker than that for the other variables. Nonetheless, we show that Stance Duration ICC in curved trajectory is not smaller than in linear trajectory, i.e. the reliability of the procedure is not affected by the shape of the trajectory. We would note that the dynamic (Peak Pressure and Peak Force) and geometric (Contact Area) variables are instead less affected by differences across sessions, while the temporal variable (Stance Duration) may be more sensitive to the admittedly minor changes in gait speed. In this light, sampling frequency may have affected the calculation. Sampling frequency is an important factor in determining the temporal resolution of the system [2], therefore small differences in Stance Duration may go undetected when using low sampling frequencies. On the contrary, the dynamic and geometric variables (Peak Pressure and Contact Area) are likely less dependent on sampling frequency.

Overall, the results indicate that a high level of reliability for insole loading variables can be obtained using Pedar-X System. This is supported by the fact that, on average, the % Err calculated from all output variables of left and right foot during the three trajectories was only 4.8%. Therefore, this instrument yielded repeatable measurements, and its use is likely to be of help in both the clinical and research setting. ICC did not always reach excellent values, but increasing the number of steps can easily overcome this problem. Further, the absence of detectable differences in ICC between the feet signifies that insole plantar devices can be used in patients with pathological gait to assess differences in foot pressure between the healthy and affected foot related to the challenge posed by curved trajectories. Our approach was to compare the reliability of the insoles during curved walking. To this aim, we deliberately used a ‘reduced’ protocol, collecting a high number of steps under identical conditions, all steps pertaining to a trajectory of constant curvature. During ADL, subjects intermingle straight and curve path spells: it would be interesting to exploit the reliability of the insole technology for assessing the walking activity during more complex and mixed trajectories, like the validated figure of 8 walkway [24].

## Conclusions

Curved trajectories are an important aspect of walking in daily life and may represent a greater challenge for impaired mobility [36]. Evaluation of turning stability may be an effective means to detect people who are at risk of falling and to determine the efficacy of medical interventions. The present study has demonstrated that reliable plantar pressure data can be collected for curved trajectories by the Pedar-X system. It can be used reliably to quantify contact area, plantar force and plantar pressure in a single region of the foot during different trajectories of gait. This measurement technique can now be used to identify the characteristics and the differences of gait in healthy subjects and in subjects with pathologies, in which curved walking may be a problem.

## Abbreviations

PF: Peak force; PP: Peak pressure; CA: Contact area; S: Stance Duration; ICC: Intraclass correlation coefficient; CI 95%: 95% confidence interval of ICC; SD: Standard deviation; LIN: Linear; CW: Clockwise; CCW: Counter-clockwise; L: Lateral region of foot; M: Medial region of foot; % Err: Percentage mean error; SEM: Standard error of measurement; MDC: Minimum detectable change; MDC_95_: at the 95% confidence level.

## Competing interests

The authors declare that they have no competing interests.

## Authors’ contributions

MG conceived the study, and participated in its design, drafting the manuscript and performed collection of data; AMT gave contributions to conception and design of the study and performed the collection and analysis of data; AN participated in design and coordination of the study and helped to draft the manuscript; MS participated in design and coordination of the study and was involved in revising it critically for important intellectual content. All authors read and approved the final manuscript.
